# The Case for Genomic Surveillance in Africa

**DOI:** 10.3390/tropicalmed10050129

**Published:** 2025-05-08

**Authors:** Rachel Ochola

**Affiliations:** School of Health, Department of Biomedical Sciences, Technical University of Kenya, Nairobi P.O. Box 52428-00200, Kenya; rachel.opiyo@gmail.com

**Keywords:** genomic surveillance, sequencing capacity, cross-sector collaboration

## Abstract

Sub-Saharan Africa has made remarkable strides in genomic surveillance, with more than 50% of countries now equipped with an in-country sequencing capacity and 98% actively contributing data to public genomic repositories. Catalyzed by the momentum of the COVID-19 pandemic, these advancements have extended far beyond SARS-CoV-2 to address a broader spectrum of public health threats, including antimicrobial resistance (AMR) and other emerging infectious diseases. This review explores these transformative achievements, identifies remaining gaps, and outlines strategic priorities for embedding genomics into the continent’s health systems. With a focus on sustainability, equity, and cross-sector collaboration, it positions Africa as a driver of global innovation in pathogen surveillance, uniquely leveraging its genetic and epidemiological diversity.

## 1. Introduction

Sub-Saharan Africa carries an enormous burden of infectious diseases, experiencing over 100 public health emergencies annually, including malaria, cholera, meningitis, Ebola, and other viral hemorrhagic fevers [1]. The emergence of antimicrobial resistance (AMR) and zoonotic spillovers further strain healthcare systems, emphasizing the need for robust surveillance mechanisms capable of providing real-time, high-resolution data to detect outbreaks, track pathogen evolution, and guide containment strategies [2,3].

Historically, Africa’s surveillance systems relied on microbiology and serology, which, while effective in pathogen detection, lacked the precision needed for real-time outbreak tracking and mutation monitoring. Precision here refers to the ability to provide consistent, timely, and detailed data, enabling the quick detection of changes in infection patterns or pathogen mutations. While accurate, traditional methods were often too slow or limited in scope to offer the rapid, continuous insights needed for effective response and decision-making. These limitations were particularly evident during the COVID-19 crises. The pandemic therefore served as a wake-up call, exposing gaps in Africa’s ability to rapidly detect and respond to emerging diseases. Subsequently, there continues to be renewed focus on strengthening the genomic surveillance capacity to improve the detection of new variants and enhance outbreak management.

Currently, more than 50% [4,5] of African Member States have established in-country genomic sequencing capabilities primarily focused on next-generation sequencing (NGS) [6]. This capacity is predominantly found in academic/research institutions and public health agencies. Moreover, with over 30 African nations adopting data protection legislation, the rise in genomic and health research is unfolding alongside the strengthening of legal safeguards for data [7].

Sequencing capacity extends beyond having equipment; it includes skilled personnel, robust data systems, and supportive policies essential for applying genomic data in public health. It plays a vital role in tackling AMR and zoonotic diseases within a One Health framework by enabling early detection, tracking transmission, identifying infection sources, predicting resistance, and guiding targeted interventions like vaccines and treatments. While many African countries have made significant progress in acquiring sequencing equipment, there still remains a pressing need to enhance and modernize the capacity for the timely management, analysis, and dissemination of pathogen sequence data. Strengthening these areas is essential to ensure that genomic surveillance efforts translate into actionable insights that inform national and regional public health decision-making.

Programs like the Africa Pathogen Genomics Initiative (PGI), led by Africa Centres for Disease Control and Prevention (Africa CDC), World Health Organization (WHO), and other global partners have been instrumental in strengthening local laboratories. This initiative focuses on equipping facilities with high-throughput sequencing platforms, advancing bioinformatics expertise, and fostering regional collaborations [8] to enhance genomic surveillance and public health responses. Africa now significantly contributes to global genomic data-sharing platforms like GISAID (the Global Initiative on Sharing All Influenza Data), exemplifying its growing role in global health security [9]. Further initiatives such as the Human Heredity and Health in Africa Bioinformatics Network (H3AbioNet) [8], the Nigerian 100K Non-Communicable Diseases-Genetic Heritage Study (NCD-GHS) [10,11], and investments in high-performance computing facilities are addressing systemic challenges in data analysis and infrastructure. These collective efforts have positioned the continent to actively combat endemic diseases like malaria [12], HIV/AIDS [13], and tuberculosis [14,15], as well as emerging threats such as AMR [16] and zoonotic spillovers [17].

High-income countries (HICs), for example in Asia, Europe, and North America, continue to provide a valuable roadmap for enhancing the impact and efficiency of genomic surveillance systems. Their comprehensive and integrated approaches highlight the potential of genomic data to shape public health policies and pandemic responses effectively. HICs have established benchmarks for success, sequencing at least 0.5% of COVID-19 cases within a 21-day turnaround time [18]. This practice ensures the rapid detection of emerging variants, enabling swift public health responses. For instance, the identification of SARS-CoV-2 variants such as Delta and Omicron within months of their emergence showcased the critical role of genomic surveillance in pandemic management [19]. One of the hallmarks of HICs’ genomic surveillance systems is their seamless integration with public health strategies. Specifically, genomic data were used not only to track variants but also to inform vaccine development, optimize treatment protocols, and tailor public health interventions, such as travel restrictions and localized containment measures [18], as seen in the responses to the COVID-19 pandemic in the United Kingdom, United States, and Australia. These actions were supported by advanced data systems that enabled the rapid analysis and real-time sharing of findings with global partners via platforms like GISAID [20,21]. Moreover, with sustainable financing models, investments in state-of-the-art sequencing technologies, bioinformatics infrastructure, and workforce development, HIC’s have ensured their systems’ resilience and scalability. Large-scale training programs in bioinformatics and genomics have created a highly skilled workforce capable of translating genomic data into actionable public health measures. Additionally, partnerships between public health institutions, academic entities, and private industry have fostered innovation and expanded the capacity for genomic applications, such as Genomics UK Consortium (COG-UK), which is supported by UK Research and Innovation, the UK Department of Health and Social Care, and the Wellcome Trust [22]; Genome Canada, a not-for-profit organization dedicated to advancing large-scale genomics and proteomics research through public–private partnerships, promoting innovation and strengthening capacity in genomic applications [23]; and the European Molecular Biology Laboratory–European Bioinformatics Institute (EMBL-EBI), which facilitates life science research and its application to medicine, agriculture, industry, and society by offering bioinformatics training to scientists across various experience levels [24]. These were exemplified by the Coronavirus Disease 2019 (COVID-19).

Ultimately, these collaborative frameworks underscore the value of cross-sectoral partnerships in accelerating the deployment of sequencing technologies, promoting equitable data sharing, and strengthening public health responses. Technology providers, global health organizations, academic institutions, governments, and pharmaceutical companies each played critical roles, from vaccine development and treatment optimization to coordinated surveillance and preparedness strategies. Such integrated networks have proven instrumental in enhancing pandemic preparedness and shaping resilient health systems.

This paper outlines how Africa can lead global innovation in pathogen surveillance. The continent has a unique advantage due to its diverse genetic makeup and epidemiological landscape, which gives it a distinct edge in addressing infectious diseases. Additionally, recent progress in strengthening genomic capabilities across the continent provides an important opportunity to enhance the detection, monitoring, and response to health threats. With these strengths, sub-Saharan Africa is well positioned to not only address global health challenges but also transform its own public health systems.

## 2. Current Perspectives in Genomic Surveillance in Sub-Saharan Africa

### 2.1. Achievements

Sub-Saharan Africa has indeed made significant strides in genomic surveillance over the past decade, with several notable achievements made during and after the pandemic. Key examples are highlighted below.

Before and during the early stages of the COVID-19 pandemic, laboratories in underserved regions across Africa relied heavily on external facilities—often located overseas—for genomic analysis. This reliance led to delayed outbreak responses and limited opportunities for local capacity building. At the time, only 7 of the 55 African Union (AU) Member States [5] had the capacity to conduct genomic sequencing and submit genomes to the Global Initiative on Sharing All Influenza Data (GISAID). However, the pandemic catalyzed a transformation in Africa’s genomic landscape, spurring initiatives to expand sequencing capacity, enhance training, and establish regional hubs within the continent for genomic surveillance. Currently this capacity stands at 53 of the 55 AU Member States in 2024 [6,25] to carry out genetic sequencing in-country. This shift was driven by collaborative efforts between African institutions, governments, global organizations, and private sector partners.

### 2.2. Key Training Initiatives and Institutional Contributions

1. World Health Organization (WHO) and Africa Centres for Disease Control and Prevention (Africa CDC) [26,27]:
  ○ Training Initiatives: WHO and Africa CDC have spearheaded efforts to build genomic capacity in Africa by providing training in genomic sequencing techniques, bioinformatics, variant tracking, and data sharing through platforms like the Integrated Genomic Surveillance and Data Sharing Platform (IGS) and support for public health laboratories across the continent. Additionally, the launch of the Integrated Genomic Surveillance for Outbreak Detection (DETECT) has supported real-time response strategies to emerging health threats.
  ○ Focus Areas: These organizations have focused on fostering practical skills for genomic data analysis, enhancing real-time outbreak response strategies, and establishing robust systems for global data sharing, resulting in the sharing of over 90,000 SARS-CoV-2 sequences, a milestone that underscores the power of collaboration in genomic surveillance compared to historical data for other diseases like HIV and influenza.
2. The Africa Pathogen Genomics Initiative (Africa PGI) [28]:
  ○ Training Initiatives: The above initiative launched by Africa CDC collaborated with regional laboratories and universities to deliver technical training on pathogen genomics, particularly for COVID-19 surveillance.
  ○ Focus Areas: Sequencing technologies, bioinformatics pipelines, and genomic data interpretation.
3. South African National Bioinformatics Institute (SANBI) [29]:
  ○ Training Initiatives: The SANBI is dedicated to building capacity in genomics and bioinformatics through training programs, the development of genome annotation methods, and fostering awareness to optimize the use of bioinformatics tools in Africa.
  ○ Focus Areas: The primary focus is on developing computational methodologies to accelerate genomics data analysis, fostering collaborations on health-related research for both communicable and non-communicable diseases, and creating specialized resources for genomics and informatics.
4. International Livestock Research Institute (ILRI) Genomics Platform [30]:
  ○ Training Initiatives: The ILRI provides both short- and long-term training programs for fellows and scientists to develop skills in modern genomic technologies applicable to life sciences and targeted research areas.
  ○ Focus Areas: The focus is on applying genomic technologies in medicine, diagnostics, public health, One Health, climate change, animal health, and agriculture to address pressing global challenges.
5. Private Sector Partnerships:
  ○ Companies like Thermo Fisher Scientific, Illumina, Oxford Nanopore, and Inqaba Biotec have either collaborated with African distributors or directly provided sequencing platforms, reagents, and training to laboratories.
6. AFROSCREEN Project [31,32]:
  ○ Training Initiatives: AFROSCREEN provides hands-on training for laboratory personnel, bioinformaticians, and public health professionals across 13 African countries. It supports skills development in genomic sequencing, data analysis, and data sharing through regional workshops, symposia, and technical exchanges.
  ○ Focus Areas: The initiative focuses on strengthening genomic surveillance systems for emerging and re-emerging pathogens, promoting data sharing through platforms like GISAID, and supporting a One Health approach to epidemic preparedness. Additional priorities include building sequencing infrastructure, enhancing workforce capacity, and establishing sentinel surveillance networks.
7. Non-governmental Organizations [33,34,35,36,37,38]:
  ○ Non-governmental organizations have played a pivotal role in advancing genomic surveillance across Africa. The Global Fund has supported laboratory infrastructure and integrated data systems to enhance disease control and pandemic preparedness. International AIDS Vaccine Initiative (IAVI) contributes to genomic research through HIV-related surveillance and vaccine development efforts. The Wellcome Trust and the Bill & Melinda Gates Foundation have provided sustained investments in research, sequencing infrastructure, and capacity building, with a strong emphasis on data equity and bioethics. The African Society for Laboratory Medicine (ASLM), a regionally based network, has been instrumental in workforce development, quality assurance, and the coordination of genomic training across national public health laboratories. Collectively, these organizations have contributed to the integration of genomics into public health systems, particularly through support aligned with One Health and epidemic preparedness frameworks.

The key institutional contributions to genomic surveillance across the continent is summarized in Table 1.

As detailed above, these institutions collectively contribute across the spectrum of genomic surveillance by enhancing laboratory capacity, expanding pathogen monitoring, advancing bioinformatics, and supporting policy frameworks. All of this is aimed at improving Africa’s readiness for current and emerging infectious diseases.

### 2.3. Genomic Advancements in Africa: Key Case Studies

Sub-Saharan Africa is advancing genomic surveillance through powerful examples of collaboration, One Health integration, and regional leadership, combining innovation with resilience. In Uganda, for example, the Uganda Genome Resource (UGR) has transformed genomic research by leveraging local infrastructure through the Uganda Virus Research Institute and international partnerships with institutions like the University of Cambridge and the Sanger Institute [39]. This collaboration, supported by bioinformatics training from the H3Africa initiative, has enabled groundbreaking studies in population genetics and genetic epidemiology. Ethiopia continues to enhance its potential of One Health integration [40], using genomic sequencing, initially developed during the COVID-19 pandemic, to combat AMR and zoonotic diseases. Despite initial challenges like limited laboratory resources, the country has proven the adaptability of these tools in addressing priority health issues. The Democratic Republic of Congo (DRC) is emerging as a regional leader by utilizing Ebola-era genomic infrastructure [41,42] to previously sequence its own SARS-CoV-2 samples, as well as from neighboring countries like Chad and Cameroon during the pandemic, and they are currently monitoring priority diseases like malaria and the current Mpox outbreak [43]. These efforts highlight Africa’s growing leadership in global health innovation. For example, African researchers have helped to identify key COVID-19 variants like Beta and Omicron. Indeed, many countries have also built a local sequencing capacity and launched regional surveillance networks, which are clear signs of leadership and rapid response in a global crisis. However, challenges remain, as earlier described: infrastructure and skilled workforce are still unevenly distributed; locally led research often lacks stable funding; and data sharing and governance frameworks need further development. Addressing these gaps is essential for a more equitable and resilient future.

### 2.4. Post-COVID-19 Advancements in Genomic Surveillance: Strengthening Africa’s Health Security

Africa CDC and Illumina expanded their partnership in November 2023 with the launch of Africa PGI 2.0 [44], building on four years of collaboration to advance genomic surveillance and public health infrastructure across the continent. The initiative broadens its focus beyond COVID-19 to include tuberculosis, malaria, cholera, and other endemic diseases. The key objectives include equipping all 55 National Public Health Institutes (NPHIs) with operational NGS capacity by 2025, increasing access to sequencing tools and reagents in 25 countries [45], and embedding genomics into public health decision-making. Since 2020, Illumina has supported this mission through the provision of sequencing platforms, reagents, and technical training [46]. These efforts are helping to shift Africa’s health systems from reactive to proactive models by enabling faster outbreak detection, variant tracking, and stronger disease control responses. The integration of genomics also lays the groundwork for precision medicine and contributes to global and regional health security.

In parallel, the African Centre of Excellence for Genomics of Infectious Diseases (ACEGID) and Illumina signed a memorandum of understanding in February 2023 to launch a genomics training academy, which aims to train 1000 African researchers and scientists in areas including NGS laboratory workflows, bioinformatics, big data analytics, and artificial intelligence (AI) applications by the end of 2025 [47]. They are also piloting a mobile “lab-in-a-container” concept to provide on-site sequencing for outbreak responses in West Africa, complementing ACEGID’s SENTINEL program focused on the early detection of pandemic threats [47].

Most recently, in March 2025, Africa CDC, the African Society for Laboratory Medicine (ASLM), and the Mastercard Foundation launched a continent-wide genomic surveillance and bioinformatics program under Phase II of the Saving Lives and Livelihoods (SLL) initiative [48]. This effort aims to improve pandemic preparedness through investments in molecular diagnostics, sequencing infrastructure, biobanking, and workforce training. The key components include the following:

- Provision of advanced laboratory equipment, reagents, and expert-led training;
- Development of a continent-wide data-sharing platform to enable real-time surveillance;
- Expansion of Africa’s genomics and bioinformatics workforce.

Together, these initiatives mark a major shift in Africa’s genomic readiness and health security architecture, reinforcing the continent’s ability to detect, monitor, and respond to both ongoing and emerging health threats.

## 3. Persistent Challenges in Genomic Surveillance

### 3.1. Infrastructure Challenges

Despite the upgrading of genomic capabilities in many African nations to date, most still face significant challenges in securing essential laboratory supplies. These include difficulties in procuring critical consumables (reagents, sequencing kits, and extraction buffers), often due to global supply chain disruptions, high costs, and limited availability from local vendors. Delays in importation processes, regulatory hurdles, and reliance on international suppliers further hinder timely access to essential materials, ultimately affecting the continuity and scalability of genomic surveillance efforts. Further challenges include the necessary bioinformatics infrastructure, e.g., online tools and other computational tools [49], and limited self-generated funding, as many African countries still heavily rely on external support rather than sustained national investments or local private sector contributions. Also, those laboratories often operate on project-specific funding, meaning that they face challenges in supporting ongoing operations once a project ends. This reliance on short-term, donor-supported initiatives jeopardizes continuity, as laboratories may struggle to retain trained personnel, maintain equipment, or continue sequencing and analysis in the absence of consistent, long-term funding. Therefore, the sustainability of genomic programs becomes severely hindered.

### 3.2. Workforce Deficits

Sub-Saharan Africa’s genomic workforce is inadequate, a glaring disparity considering the continent’s approximately 1.2-billion–person market [50] share of the global population. This gap is concerning given the increasing reliance on bioinformatics for genomic analysis and outbreak management. Programs like H3 Africa [10] have successfully trained early-career bioinformaticians, but retention remains a challenge. It has been noted that 20,000 highly educated professionals, of whom comprise up to 30% trained scientists, leave their roles annually, often seeking better-funded or more stable opportunities outside the continent [51].

### 3.3. Analysis

In addition to retention issues, a significant misalignment between academic training and laboratory needs [52] contributes to skill mismatches across the genomic workforce. Indeed, many educational programs prioritize theoretical instruction, offering limited exposure to applied genomic and bioinformatics techniques. Furthermore, the absence of clear career pathways and professional growth opportunities discourages mid-career professionals from remaining in or advancing within the field.

### 3.4. Proposed Solutions

1. Expand undergraduate and postgraduate programs in bioinformatics and molecular biology to comprise competency-based curricula emphasizing practical applications and on-the-job training. While, historically, partnerships with international institutions have contributed to knowledge transfer in genomic surveillance, strengthening intra-African collaboration is essential for sustainability.
2. Introduce competitive incentives, such as research grants, tax exemptions for scientists, and subsidized housing to retain skilled professionals [53]. Recognizing contributions through awards and career advancement opportunities may also improve retention.
3. Establish and/or further strengthen African regional Centers of Excellence as core pillars of the continent’s genomic surveillance ecosystem, with a focus on capacity building, workforce development, and regional data-sharing leadership. This would promote self-reliance and ensure contextual relevance by tailoring efforts to the continent’s unique epidemiological, logistical, and policy environments.

### 3.5. Ethical and Data Governance Issues

In recent years, a significant proportion of genomic data from African laboratories have been analyzed in external facilities, prompting debates around data sovereignty and equitable access. Although platforms like GISAID facilitate global data sharing, many sub-Saharan African countries still lack comprehensive policies that safeguard participant privacy while ensuring the fair use of shared data. This imbalance has raised critical questions about benefit-sharing and the autonomy of local institutions. The evolving data protection landscape across Africa further complicates this issue. While national frameworks often share foundational principles—such as informed consent, data security, and adequacy for cross-border transfers—their implementation remains inconsistent. A review of data protection policies across 12 African countries revealed a fragmented legal approach that required researchers to adapt to diverse and often complex compliance pathways [7]. The lack of standardized data-sharing agreements has subsequently contributed to delays in data access, reduced participation in regional databases, and hindered real-time responses to outbreaks. For instance, during the early phases of the COVID-19 pandemic, concerns over regulatory approvals and ownership delayed the release of genomic data in several countries. Similar barriers were observed during previous outbreaks such as Ebola and yellow fever, where limited data sharing impeded genomic tracking and regional coordination. These challenges underscore the urgent need for coherent, continent-wide data governance strategies that uphold participant rights, enable rapid and responsible data sharing, and ensure that African institutions remain central to the generation and use of genomic data.

### 3.6. Proposed Solutions

1. Standardize data-sharing frameworks to ensure participant privacy while enabling global collaboration.
2. Build local capacity for bioinformatics analysis [53,54] to reduce dependency on external laboratories and ensure data sovereignty.
3. Encourage the government to develop policies that balance data sovereignty with equitable sharing whilst ensuring that their own researchers and institutions benefit from collaborations and maintaining participant privacy.
4. Public education campaigns should highlight the role of genomics in improving health outcomes, with a bid to foster trust and encouraging participation in genomic research.

## 4. Regional Disparities in Genomic Surveillance

Despite clear WHO recommendations urging countries to develop national genomic strategies [55], efforts to expand sequencing beyond SARS-CoV-2 remain limited. Progress has been uneven, largely due to persistent gaps in infrastructure, workforce capacity, funding, and governance. While many solutions have been proposed, translating global guidance into actionable, country-led implementation requires sustained political will, stronger coordination, and greater domestic investment.

These disparities are not merely technical; they reflect deeper historical, economic, and political dynamics that have shaped regional development across the continent. Southern sub-Saharan Africa, and particularly South Africa, benefits from a long-standing culture of research investment, a strong academic ecosystem, and consistent government support for biomedical innovation. In contrast, Central and parts of West Africa face systemic underinvestment in science and technology, often exacerbated by political instability, limited domestic financing, and weak health governance structures.

Donor-driven development models have further reinforced these regional imbalances [56]. Countries with stronger visibility in global health programs and established international partnerships have attracted greater sequencing investments. Meanwhile, others—despite having equally pressing needs—remain under-resourced and overlooked. Digital infrastructure and access to bioinformatics training also contribute to this widening gap. As some regions advance with integrated data systems, others continue to rely on manual processes or external partners, limiting their autonomy in genomic surveillance.

Addressing these inequities goes beyond simple technology transfer. It demands long-term region-sensitive investments. This includes building strong leadership and optimizing resource allocation through regional mapping to reduce redundancy and address gaps. Additionally, strengthening intra-African research and training networks, and having strong political commitment, will help to build self-sustaining genomic ecosystems across the continent.

*Strategies to Narrow Regional Gaps*

1. Establish Regional Sequencing Resource Hubs

Countries with stronger infrastructure (e.g., South Africa, Nigeria, Kenya, Senegal) can serve as technical anchors, supporting neighboring countries through sample sharing, mobile laboratories, and mentorship.

2. Promote Regional Funding Mechanisms

Encourage shared funding pools, e.g., under Africa CDC and African Union Development Agency-New Partnership for Africa’s Development (AUDA-NEPAD) that prioritize equity across less-resourced regions, reducing over-reliance on external donors.

3. Harmonize Data-Sharing and Policy Frameworks

Develop cross-border agreements to standardize bioethics, data ownership, and legal compliance, facilitating regional collaboration.

4. Target Political Will and Governance Gaps

Advocate for national governments in underperforming regions to prioritize genomics in health strategy and allocate domestic budgets accordingly.

5. Foster South–South Knowledge Exchange

Formalize platforms for African experts to train and support peers across borders, leveraging regional expertise over imported models.

*The Importance of Strengthening Surveillance in Sub-Saharan Africa*

There remain immense public health benefits that can be realized through genomic surveillance. This encompasses the real-time tracking of mutations, informing targeted interventions. For example, recently, next-generation sequencing of malaria pathogens in East Africa identified drug-resistant strains, guiding treatment protocols [15]. Additionally, with timely genomic surveillance, the economic benefits to a country would be positive; costly outbreaks could be prevented. Indeed, a study by Huber et al. [57] estimated that early containment of an Ebola outbreak could save USD 53.19 billion (2014) in healthcare and further economic losses. Scaling such efforts would, however, have to occur continent-wide to transform the continent’s outbreak response capabilities.

## 5. Future Directions and Recommendations

**Figure 1:**
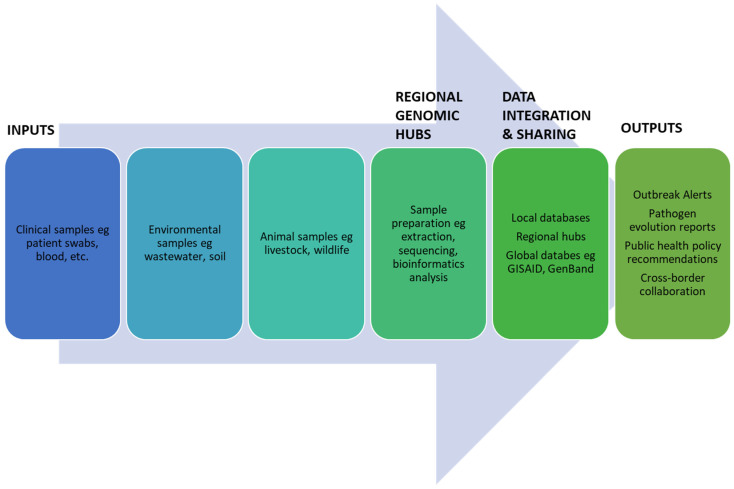
Multi-pathogen genomic framework for regional hubs.

Figure 1 outlines a proposed framework for integrating multi-pathogen genomic surveillance within regional hubs. Starting with sample inputs from clinical, environmental, and animal sources, the framework emphasizes the central role of regional genomic hubs equipped with bioinformatics tools for processing data. These hubs generate actionable outputs, such as real-time outbreak alerts, pathogen evolution reports, and policy development insights. By visualizing the stepwise process, the flowchart underscores the importance of regional integration and multi-pathogen surveillance for optimizing resources and enhancing Africa’s outbreak preparedness.

Therefore, the important components would comprise the following:

- Inputs: The framework begins with sample collection from various sources—clinical, environmental, and animal—to ensure comprehensive pathogen monitoring.
- Regional Genomic Hub: Centralized facilities process and analyze the received samples, focusing on sequencing and bioinformatics to derive actionable insights.
- Data Integration and Sharing: The processed data are integrated into local and global databases, ensuring accessibility and collaboration.
- Outputs: The system then generates outputs critical for public health, such as real-time outbreak alerts, mutation tracking, and recommendations for containment strategies.

### Recommendations

To sustain and further advance genomic surveillance in Sub-Saharan Africa, the following recommendations are essential:

1. Strengthening Infrastructure and Expanding African Regional Hubs:
  - Develop more regional genomic surveillance hubs to address disparities and improve access across underserved regions.
  - Consolidate and optimize existing sequencing infrastructure by supporting technology upgrades, expanding geographic coverage, and strengthening data integration and analysis capacity. Specifically, to enhance the accuracy and speed of genomic insights, it is critical to invest in locally adapted bioinformatics tools and establish centralized, ethically governed data-sharing platforms. Initiatives like H3ABioNet [8] and Africa CDC’s [26,27] data strategy are laying the groundwork for ethically governed and interoperable genomic data platforms in Africa. However, their long-term success will depend on investments in local capacity, common data standards, and inclusive governance frameworks that safeguard African interests.
  - Implement multi-pathogen surveillance frameworks that integrate clinical, environmental, and animal health data under a One Health approach, ensuring the comprehensive monitoring of zoonotic diseases, AMR, and other emerging threats.
2. Workforce Development and Retention:
  - Establish undergraduate, postgraduate, and professional training programs focusing on applied genomics, bioinformatics, and molecular epidemiology with competency-based curricula tailored to local needs.
  - Provide competitive retention incentives, including research grants, career advancement opportunities, and non-monetary benefits such as housing subsidies to reduce the loss of skilled professionals.
  - Foster mentorship and networking opportunities through partnerships with global institutions, enabling knowledge transfer and sustained professional development.
3. Promoting Sustainability Through Financing and Policy Support:
  - Secure long-term funding mechanisms through public–private partnerships and international collaborations to reduce reliance on donor-specific funding.
  - Encourage governments to allocate national budgets toward genomic surveillance as a public health priority, ensuring the operational sustainability of laboratories and workforce initiatives.
  - Advocate for tax incentives for private companies investing in genomic research and innovation.
4. Enhancing Data Governance and Collaboration:
  - Develop robust policies to balance data sovereignty with equitable data sharing, ensuring that African researchers benefit from global collaborations while protecting participant privacy.
  - Strengthen regional and international collaborations through platforms such as GISAID, ensuring the timely sharing of genomic data to guide public health responses.
  - Build interoperable data systems across countries and regions to enable seamless data integration, supporting the real-time tracking of outbreaks and emerging threats.
  - Encourage partnerships with global consortia to increase Africa’s representation in genomic studies while fostering knowledge transfer.
5. Fostering Innovation and Research:
  - Promote research into Africa-specific pathogens and health challenges, leveraging the continent’s unique genetic and epidemiological diversity to guide the development of tailored vaccines and treatments.
  - Support translational research that bridges the gap between genomic data generation and its application in public health policy and clinical practice.
  - Encourage the use of genomic tools in monitoring vaccine effectiveness and drug resistance, ensuring evidence-based interventions.
6. Community Engagement and Advocacy:
  - Engage communities in the importance of genomic surveillance to build public trust and promote participation in research and outbreak management initiatives.
  - Raise awareness among policymakers about the economic and public health benefits of investing in genomics.
  - Incorporate genomic education into broader health literacy programs to foster societal understanding and support for genomic initiatives.

## 6. Conclusions

The COVID-19 pandemic no doubt significantly reshaped Africa’s genomic landscape, catalyzing widespread knowledge transfer and capacity building. While many initiatives have laid the groundwork for sustainability—through in-country sequencing, workforce training, and integration into health systems—continued investment and support are essential to preserve and build on these gains. It is imperative that Africa now leverages these lessons, harnessing its unparalleled genetic diversity and recent advancements in genomic capacity to shape a more resilient and self-reliant public health future. This will position the continent as an indispensable player in the global genomic landscape. This diversity—the richest of any continent—holds immense potential for groundbreaking discoveries in disease etiology, therapeutic development, and precision medicine. Sub-Saharan Africa’s progress in genomic surveillance highlights its resilience and capacity for innovation, as the region transitions from reliance on external facilities to building in-country capacities and centers of excellence. These advancements have laid a robust foundation for transformative public health practices, underscoring the continent’s ability to adapt and lead in the genomic era. However, systemic challenges, including infrastructure gaps, disparities in access to resources, and a shortage of skilled personnel, threaten to hinder progress unless urgently addressed.

To fully realize these benefits, a holistic and sustainable approach is essential. Addressing infrastructure gaps will require strategic investments in sequencing facilities, bioinformatics platforms, and computational resources. Workforce development must also be prioritized, with scalable training programs in genomics and bioinformatics to cultivate a skilled cadre of scientists and public health professionals who can translate genomic insights into actionable outcomes. Equally important is the establishment of innovative financing models that integrate government funding, international support, and private sector investment to ensure the long-term sustainability of genomic surveillance systems. Sustainable financing mechanisms, akin to those in HICs, will be vital for maintaining and expanding genomic surveillance systems. Finally, setting benchmarks for timely sequencing and data analysis, such as the 21-day turnaround standard, can ensure that sub-Saharan Africa remains at the forefront of pathogen surveillance and outbreak response.

Collaboration remains pivotal in overcoming these challenges. Regional and global partnerships have already demonstrated their effectiveness in amplifying Africa’s genomic efforts. Initiatives like the Africa PGI and platforms such as GISAID exemplify the potential of partnerships to facilitate knowledge exchange, resource sharing, and coordinated cross-border responses. Expanding these collaborations to include interdisciplinary teams and community-driven approaches will further strengthen Africa’s genomic ecosystem and ensure that its benefits are equitably distributed.

Genomic surveillance has the potential to revolutionize public health systems across Africa by enabling real-time pathogen tracking, fostering the development of targeted vaccines and treatments and strengthening responses to both emerging and endemic health threats. In a region facing a disproportionate burden of global infectious diseases, compounded by the challenges of AMR and zoonotic spillovers, integrating genomic data into health systems is essential. This approach enhances the ability to monitor pathogen evolution, detect outbreaks earlier, and implement rapid, evidence-based interventions, bolstering health security within Africa while contributing to global efforts in pandemic prevention and control. Africa’s expanding genomic capacity not only equips it to address diseases like malaria, HIV/AIDS, and tuberculosis but also positions the continent as a leader in tackling global health challenges. With strategic investments, collaborative partnerships, and a focus on integrating genomics into health systems, Africa stands poised to transform its healthcare landscape, secure better health outcomes for its people, and set a global standard for equitable and innovative public health solutions. The case for genomic surveillance on the African continent is not merely a call to action; it is a unique opportunity to redefine global health equity and innovation.

## Figures and Tables

**Table 1:** Summary of key institutional contributions to genomic surveillance in sub-Saharan Africa.

Institution	Description	Contributions to Genomic Surveillance
1. World Health Organization (WHO) and Africa Centres for Disease Control and Prevention (Africa CDC)	Global and regional public health authorities leading efforts in coordination, policy guidance, and capacity building.	Provide strategic leadership and technical guidance on genomic surveillance standards. Support integration of genomics into disease surveillance systems, promote data sharing, and facilitate capacity building across African nations.
2. Africa Pathogen Genomics Initiative (Africa PGI)	A flagship initiative to build and expand pathogen genomics capacity across the African continent.	Established operational NGS capacity in all 55 AU Member States by 2025. Focus on integrated surveillance for human, animal, and environmental health (One Health). Distributes sequencing platforms and reagents, provides training, and enhances data sharing.
3. South African National Bioinformatics Institute (SANBI)	A leading African institute in bioinformatics research and training.	Supports bioinformatics training and capacity building. Provides technical support in data analysis and genome assembly. Develops and maintains analysis pipelines to strengthen regional bioinformatics networks.
4. International Livestock Research Institute (ILRI) Genomics Platform	An international research organization focusing on livestock and zoonotic disease research.	Conducts genomic surveillance of zoonotic pathogens. Supports One Health surveillance through sequencing of animal pathogens, contributing to early detection and control of zoonotic disease threats in Africa.
5. Private Sector Partnerships (e.g., Thermo Fisher Scientific, Illumina, Oxford Nanopore, Inqaba Biotec)	Global biotechnology companies providing advanced genomic sequencing technologies, reagents, and analytical tools.	Supply sequencing platforms, reagents, and bioinformatics solutions critical for genomic surveillance; facilitate technology transfer, training, and infrastructure development; collaborate with public health institutions to enhance sequencing capacity and rapid pathogen detection across Africa.
6. AFROSCREEN	Multi-country initiative coordinated by French research institutions (ANRS|MIE, Institut Pasteur, and IRD) in partnership with 13 primarily Francophone African countries. Focuses on strengthening genomic surveillance systems through regional collaboration, infrastructure support, and workforce development.	Builds sequencing capacity through provision of equipment and training. Enhances workforce skills in genomics and bioinformatics, supports data sharing via platforms like GISAID, promotes a One Health approach, and improves outbreak response through regional coordination and sentinel surveillance.
7. Non-governmental Organizations		
ASLM	Africa-based; specializes in laboratory systems and accreditation across the continent.	Regional training, laboratory network coordination, quality assurance, and workforce development.
Bill & Melinda Gates Foundation (BMGF)	Focused on high-burden diseases; combines research, delivery, and system support	Genomic epidemiology and modeling domain aims to expand access to genomic surveillance in low- and middle-income countries and enhance disease modeling and mapping through global collaboration. Strengthens laboratory systems and funds sequencing and diagnostic tools for malaria, TB, and COVID-19.
Global Fund	Invests in diagnostic infrastructure for HIV, TB, and malaria; integrates genomics into disease surveillance.	Support for genomic surveillance is enhancing disease control efforts and pandemic preparedness by improving laboratory infrastructure and integrated data systems for faster detection and response.
IAVI	Focused on translational science and vaccine development for HIV and emerging infections.	HIV-related genomic surveillance and vaccine research; supports sequencing-related data systems.
Wellcome Trust	Major philanthropic funder supporting public health genomics and academic research collaborations.	Funds research, genomics capacity, training, and bioethics programs across Africa.

## Data Availability

No new data were created or analyzed in this study. Data sharing is not applicable to this article.
